# Review of the health benefits of habitual consumption of miso soup: focus on the effects on sympathetic nerve activity, blood pressure, and heart rate

**DOI:** 10.1186/s12199-020-00883-4

**Published:** 2020-08-31

**Authors:** Koji Ito

**Affiliations:** Department of Clinical Laboratory, Japan Community Healthcare Organization, Kyushu Hospital, 1-8-1, Kishinoura, Yahatanishi-ku, Kitakyushu, 806-8501 Japan

**Keywords:** Miso, Hypertension, Sympathetic nerve activity, Blood pressure, Heart rate

## Abstract

High salt intake increases blood pressure, and dietary salt intake has been clearly demonstrated to be associated with hypertension incidence. Japanese people consume higher amounts of salt than Westerners. It has been reported that miso soup was one of the major sources of daily salt intake in Japanese people. Adding salt is indispensable to make miso, and therefore, in some cases, refraining from miso soup is recommended to reduce dietary salt intake. However, recent studies using salt-sensitive hypertensive models have revealed that miso lessens the effects of salt on blood pressure. In other word, the intake of miso dose not increase the blood pressure compared to the equivalent intake of salt. In addition, many clinical observational studies have demonstrated the absence of a relationship between the frequency of miso soup intake and blood pressure levels or hypertension incidence. The mechanism of this phenomenon seen in the subjects with miso soup intake has not been fully elucidated yet. However, in basic studies, it was found that the ingredients of miso attenuate sympathetic nerve activity, resulting in lowered blood pressure and heart rate. Therefore, this review focused on the differences between the effects of miso intake and those of the equivalent salt intake on sympathetic nerve activity, blood pressure, and heart rate.

## Introduction

Elevated blood pressure is an important reason for increased morbidity and mortality [1, 2]. Both basic and clinical studies have shown that high salt intake increases the blood pressure [3–5]. A well-known study called The INTERSALT study revealed that dietary salt intake is related to the blood pressure level, after adjusting for confounding factors [6]. In addition, high salt intake is associated with a higher risk of cardiovascular disease [7], and dietary changes, which included reduction in salt intake, were reported to be associated with lower blood pressure. Therefore, reducing salt intake has been established as a standard method to prevent and treat hypertension [8–10], with patients instructed to refrain from salt intake for the prevention and treatment of hypertension.

Recently, the role of sympathetic nerve activity (SNA) in the pathogenesis of salt-induced hypertension has gained emphasis [11, 12]. The sympathetic nervous system, which modulates SNA, is well known to play an important role in the regulation of blood pressure and heart rate [13, 14]. Although the precise mechanisms remain unclear, several factors are reported to contribute to salt-induced sympathoexcitation. For example, in Dahl salt-sensitive rats (Dahl-S rats) and spontaneously hypertensive rats (SHR), which are known to be salt-sensitive hypertensive rats, higher dietary intake of salt increased the sodium concentration in the cerebrospinal fluid (CSF[Na]) [15]. The increase in CSF[Na] activates the neurons located in the cardiovascular center, resulting in sympathoexcitation [16]. Brain mineralocorticoid receptor (MR), epithelial sodium channels, and angiotensin type 1 receptor (AT1R) also contribute to salt-induced sympathoexcitation [17–21]. The activation of these pathways occurs only in salt-sensitive models, but not in salt-resistant models [15]. In contrast, intra-cerebroventricular (ICV) infusion of high-sodium artificial CSF (aCSF) increases SNA, blood pressure, and heart rate, even in salt-resistant models [22]. Collectively, these results indicate that the sympathetic nervous system plays an important role in salt-induced hypertension by modulating SNA. In addition, it has been clarified that an increase in SNA, particularly in renal SNA, plays an important role in salt-induced hypertension [23].

Compared with the Westerners, Japanese people consume higher amounts of salt [24] with miso soup and pickles being the major sources of daily salt intake in the Japanese [25]. Miso is a traditional Japanese soybean paste made from fermented soybeans. It is often prepared into a soup and is one of the major components of Japanese-style cooking. One serving of miso soup contains 1–2 g of salt, and the addition of salt is indispensable to miso preparation. Therefore, in some cases, it is recommended to refrain from miso soup to reduce dietary salt intake. However, recent studies using salt-sensitive hypertensive models have demonstrated that the intake of miso does not increase SNA, blood pressure, and heart rate compared with the equivalent intake of salt. Therefore, this review focused on the differences between the effects of miso intake and those of the equivalent salt intake on SNA, blood pressure, and heart rate.

## Expectations for soybean products, a major constituent of Japanese-style diet

International interest in the Japanese-style diet has increased because of its health benefits. In fact, some reports have demonstrated that a Japanese-style diet reduces total mortality [26] and is associated with a longer survival time [27]. A recent qualitative systematic review study has evaluated the Japanese-style diet and has categorized its dietary characteristics [28]. This study segregated Japanese dietary constituents into 16 categories and revealed that the top 3 categories are soybeans/soybean-derived products, seafood, and vegetables, followed by rice and miso soup. Miso is also a soybean product. Therefore, soybean products are considered to contribute to the health benefits of Japanese-style diets.

Specifically, miso is a fermented soybean product. Fermentation is reported to improve the quality of soybeans, resulting in increased digestibility, enhanced nutrition, and increased isoflavone content [29]. Therefore, fermented soybean products are expected to have greater health benefits than nonfermented soybean products. In fact, a recent study has revealed that the intake of fermented soy products, but not nonfermented soy products, is inversely associated with developing high blood pressure in subjects with normal blood pressure [30]. Because miso, which is the focus of this review, is a traditional Japanese fermented soybean product, it is expected to have a significant impact on suppressing the development of high blood pressure.

## Miso intake in basic studies

Many studies have revealed that habitual dietary intake of miso soup can prevent the worsening of hypertension caused by high salt intake in genetically salt-sensitive models. One study reported that the habitual dietary intake of miso soup attenuates the salt-induced blood pressure elevation compared with the equivalent intake of salt in Dahl-S rats, with the magnitude of blood pressure reduction caused by habitual dietary intake of miso soup reaching 35 mmHg [31]. Another study reported that the estimated decreases in blood pressure caused by habitual dietary intake of miso corresponded to 30% decrease in salt loading [32]. This study also revealed the natriuresis and diuresis through the dopamine system by habitual dietary intake of miso. In addition, the attenuation of the salt-induced blood pressure elevation observed in habitual dietary intake of miso soup was also demonstrated in stroke-prone SHR, which are also genetically salt-sensitive rats [33]. Miso intake was also confirmed to be associated with alleviation of organ damage and have protective effects against stroke [32–34].

**Fig. 1:**
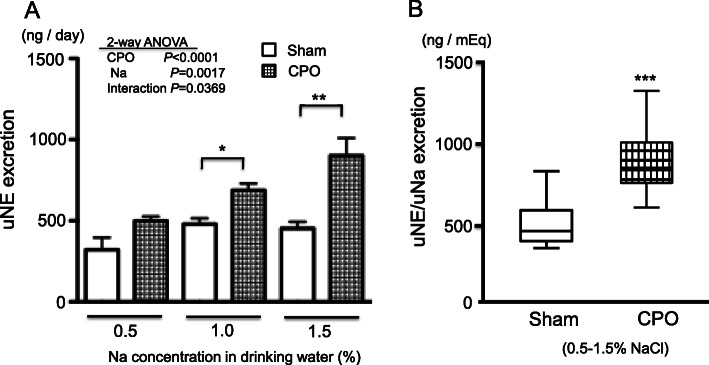
**a** SNA evaluated on the basis of uNE excretion in each group. *p < 0.05, **p < 0.01 Sham versus CPO, *n* = 8 for each. Open bar graphs indicate Sham; square-block bar graph indicates CPO. **b** The ratio of uNE/uNa excretion, as a marker of SNA in response to salt intake. ***p < 0.001 Sham versus CPO, *n* = 24 for each. Open bar graphs indicate Sham; square-block bar graph indicates CPO. SNA, sympathetic nerve activity; Sham, sham-operated control mice; CPO, chronic pressure overload mice; uNE, 24-h urinary norepinephrine; uNa, 24-h urinary sodium

**Fig. 2:**
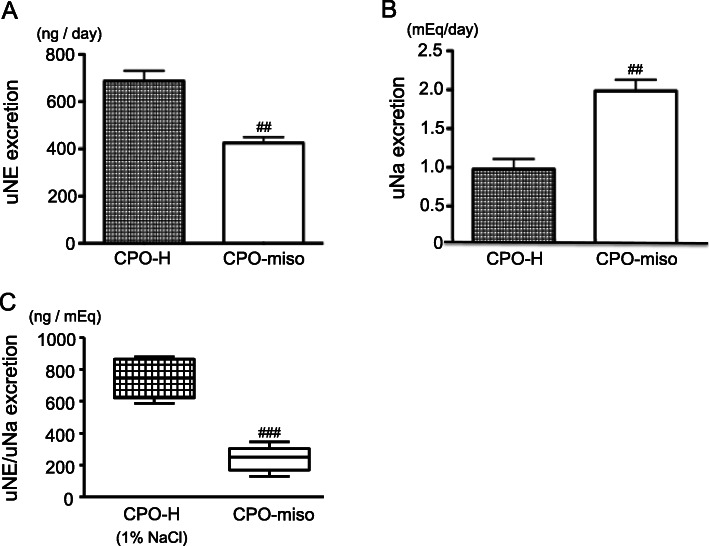
**a** SNA evaluated on the basis of uNE excretion in each group. ^##^*p* = 0.001, CPO-H versus CPO-miso, *n* = 8 for each. Open bar graphs indicate CPO-miso; square-block bar graph indicates CPO-H. **b** Na intake in terms of uNa excretion in each group. ^##^*p* = 0.001, CPO-H versus CPO-miso, *n* = 8 for each. Open bar graphs indicate CPO-miso; square-block bar graph indicates CPO-H. **c** The ratio of uNE/uNa excretion, as a marker of SNA in response to salt intake. ^###^*p* < 0.001, CPO-H versus CPO-miso, *n* = 8 for each. Open bar graphs indicate CPO-miso; square-block bar graph indicates CPO-H. SNA, sympathetic nerve activity; CPO-H, chronic pressure overload mice with high salt water; CPO-miso, chronic pressure overload mice with miso soup; uNE, 24-h urinary norepinephrine; uNa, 24-h urinary sodium

These effects observed in habitual dietary intake of miso soup were also confirmed using non-genetically salt-sensitive model. Non-genetic salt-sensitivity is acquired via abdominal aortic banding in mice [17]. The aortic banding causes chronic pressure overload (CPO) in the left side of the heart, resulting in left ventricular hypertrophy. In mice with CPO (CPO-mice), the brain hypothalamic MR- AT1R pathway gets activated [18–20]. Activation of this pathway combined with high salt intake leads to an increase in SNA, concomitant with further activation of this pathway. Figure 1 shows SNA evaluated on the basis of 24-h urinary norepinephrine (uNE) excretion [35], which increased according to the salt concentration of drinking water in CPO-mice but not in sham-operated control mice (Sham-mice) (Fig. 1a). The ratio of uNE/24-h urinary sodium (uNa) excretion, indicating uNE excretion per uNa excretion of 1 mEq, as a marker of SNA in response to salt intake was greater in CPO-mice than in Sham-mice (Fig. 1b). These results demonstrated that CPO-mice have acquired non-genetic salt sensitivity. In contrast, habitual dietary intake of miso soup suppressed the activation of the brain hypothalamic MR-AT1R pathway in CPO-mice, even though miso soup contained a measurable amount of salt [35]. In CPO-mice with miso soup consumption, uNE excretion was low (Fig. 2a), whereas uNa excretion was high (Fig. 2b) when compared with CPO-mice with high-salt water consumption. Therefore, the ratio of uNE/uNa excretion ratio was found to be lower in CPO-mice with miso soup consumption than in those with high-salt water consumption (Fig. 2c). In this study, heart rates were also lower in CPO-mice fed with miso soup than in CPO-mice fed with high salt water (499 ± 11 vs 535 ± 14 bpm) [35]. These results show that habitual dietary intake of miso soup can attenuate salt-induced sympathoexcitation and blood pressure elevation, despite the increased amount of salt intake.

## Miso intake in clinical studies

Increased miso soup intake is expected to cause hypertension, particularly in subjects with salt-sensitivity, because of increased salt intake. However, previous studies have demonstrated that the frequency of miso soup intake was not associated with high levels of blood pressure. One study reported that two daily servings of miso soup for 3 months did not affect the blood pressure of subjects with normotension or stage I hypertension [36]. In addition, a community-based prospective study reported that an increased intake of miso soup is associated with a reduced incidence of cardiovascular disease [37]. Intake of fermented soy products, such as miso and natto, was reported to be inversely associated with the development of high blood pressure in Japanese adults with normotension [30]. The results of these observational studies show that habitual miso soup intake has no effect on the levels of blood pressure or hypertension incidence in subjects with normotension or mild hypertension.

**Fig. 3:**
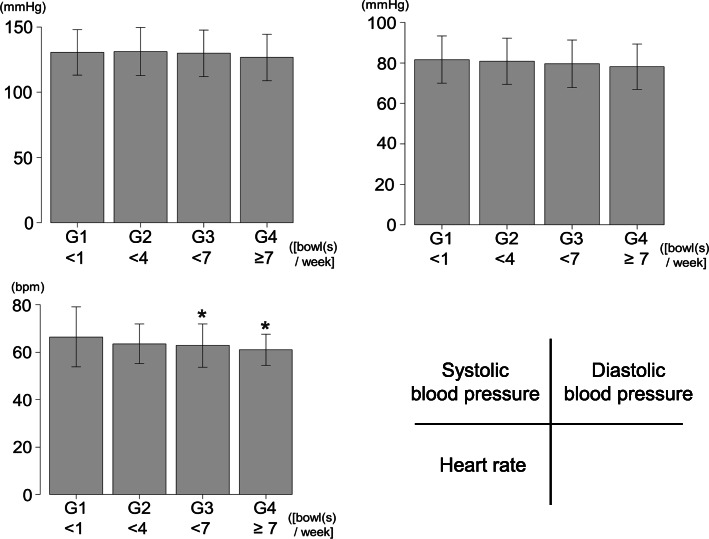
Blood pressure levels and heart rates in each group. G1, Group 1 (< 1 bowl of miso soup/week); G2, Group 2 (< 4 bowls of miso soup/week); G3, Group 3 (< 7 bowls of miso soup/week); G4, Group 4 (≥ 7 bowls of miso soup/week). *p < 0.05 versus G1

Recently, it was reported that heart rate in subjects with habitual miso soup intake was lower than in those without it [38]. Figure 3 shows the levels of blood pressure and heart rate in each group, with four groups stratified according to the frequency of miso soup intake. There was no association between the frequency of miso soup intake and the levels of blood pressure. However, the heart rate of subjects who reported a high frequency of miso soup intake was lower than that of subjects who reported a lower frequency of miso soup intake. A multivariable analysis adjusting for a priori identified covariates revealed that the frequency of miso soup intake independently affects heart rate but not blood pressure [38].

It is well known that the occurrence of sudden death due to acute cardiovascular diseases, such as acute myocardial infarction and cerebral infarction, increases in winter [39, 40]. The prevalence of hypertension also increases in winter [41] and the seasonal variations in SNA contribute to the increase in blood pressure in winter [42]. High SNA is well known to be associated with poor prognosis resulting from worsening cardiovascular disease [43–45]. Therefore, attenuation of SNA, particularly in the winter, is thought to have a significant impact on the prevention and treatment of cardiovascular diseases. A study evaluated the relationship between habitual miso soup intake and the levels of blood pressure or heart rate of Japanese adults and categorized the study into four seasons [46]. The heart rate of the participants who seldom consumed miso soup was higher in winter than in the other seasons. However, the heart rate of those who frequently had miso soup was not high in winter compared with that in the other seasons. Heart rate is modulated by autonomic nerve balance and is correlated with SNA; thus, it is a useful marker of SNA [47]. Therefore, habitual miso soup intake may be useful for preventing cardiovascular disease via the attenuation of enhanced SNA in winter.

## Effects of miso on sympathetic nerve system

**Fig. 4:**
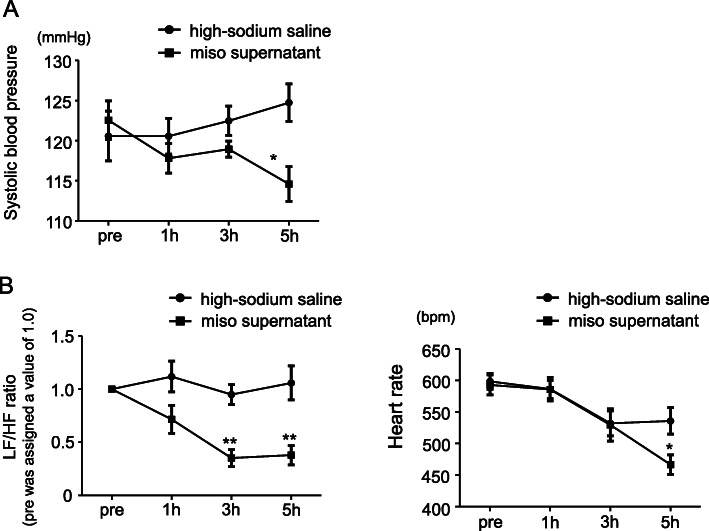
**a** Systolic blood pressure in normal mice after IP injection of high-sodium saline or miso supernatant. *n* = 5 for each. *p < 0.05 high-sodium saline versus miso supernatant. **b** LF/HF ratio (left) and heart rate (right) in normal mice evaluated via ECG. The LF/HF ratio before the injection was assigned a value of 1.0. *n* = 9 for each. *p < 0.05, **p < 0.01 high-sodium saline versus miso supernatant. IP, intraperitoneal; LF, low frequency power of heart rate variability; HF, high frequency power of heart rate variability

**Fig. 5:**
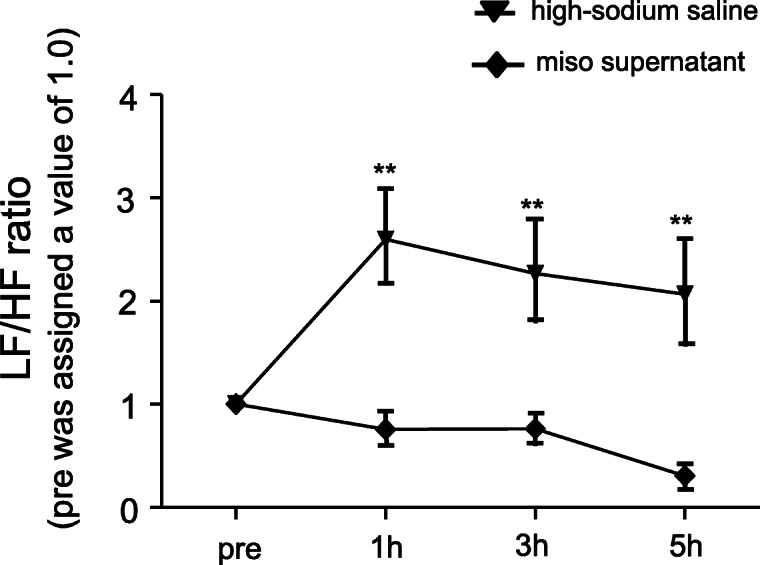
LF/HF ratio in CPO-mice evaluated via ECG. The LF/HF ratio before the injection was assigned a value of 1.0. *n* = 5 for each. **p < 0.01 high-sodium saline versus miso supernatant. LF, low frequency power of heart rate variability; HF, high frequency power of heart rate variability

The relationship between miso intake and SNA has been clearly demonstrated in a previous study [48]. As shown in Fig. 4, intraperitoneal (IP) injection of high-sodium saline did not change the blood pressure in mice without salt-sensitivity (i.e., normal mice). However, the IP injection of miso supernatant with sodium level equivalent to high-sodium saline decreased the blood pressure (Fig. 4a). In this study, heart rate was evaluated using electrocardiogram (ECG), and SNA was also evaluated using ECG time-frequency analysis. As shown in Fig. 4b, the low frequency (LF)/high frequency (HF) ratio and the heart rate did not change on IP injection of high-sodium saline, but both the LF/HF ratio and heart rate decreased on IP injection of miso supernatant. Low LF/HF ratio indicates low SNA. Therefore, systemic administration of miso supernatant decreases SNA in normal mice. As shown in Fig. 5, in CPO-mice (i.e., non-genetically salt-sensitive mice), the LF/HF ratio increased on IP injection of high-sodium saline, indicating salt-induced sympathoexcitation. These results are consistent with the salt sensitivity in CPO-mice. Interestingly, IP injection of the miso supernatant did not increase the LH/HF ratio even when the sodium level was the same as the high-sodium saline. These results indicate the cancelation of salt-induced sympathoexcitation by systemic administration of miso supernatant. In fact, a recent study demonstrated that the decrease in blood pressure due to miso intake in subjects with high-normal blood pressure or stage I hypertension may be caused by deactivation of the adrenergic nervous system [49].

**Fig. 6:**
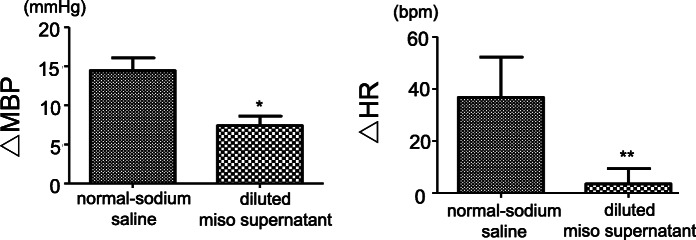
Changes in mean blood pressure (left) and heart rate (right) after high-sodium saline ICV infusion. *n* = 5 for each. *p < 0.05, **p < 0.01 high-sodium saline versus miso supernatant. ICV, intra-cerebroventricular

SNA is modulated by the sympathetic nervous system located in the brain. The possibility that the ingredients of miso work directly in the brain has also been demonstrated in a previous study [48]. Increase in CSF[Na] is known to increase blood pressure and heart rate [22]. As shown in Fig. 6, the degree of increases in blood pressure and heart rate produced by the ICV infusion of high-sodium aCSF was smaller in mice pre-treated with diluted miso supernatant (0.14 M sodium) than in those pre-treated with normal-sodium saline (0.14 M sodium) [48]. The results of this study indicate that the ingredients of miso work in the brain to modulate SNA and to attenuate salt-induced blood pressure and heart rate elevation.

The mechanisms involved in attenuated salt-induced sympathoexcitation following habitual dietary intake of miso soup have not been fully understood. As mentioned above, miso is a soybean product. Therefore, isoflavone may be involved in the inhibitory effects of miso on SNA, blood pressure, and heart rate [50, 51]. The results from previous studies have revealed that inhibition of the renin-angiotensin system (RAS) plays an important role in the effects caused by miso intake. The RAS in the brain modulates SNA and contributes to the pathogenesis of hypertension [52, 53]. Interestingly, fermented soybeans, including miso, are known to block the angiotensin-converting enzyme (ACE) activity [54, 55]. The active ingredient of miso that has ACE inhibitory activity was reported to be a small peptide [33]. Therefore, such small peptides with anti-ACE inhibitory effects might work directly in several organs, including the brain. In fact, it was revealed that the habitual intake of miso soup decreases AT1R expression in the brain of CPO-mice [35]. Therefore, inhibition of brain RAS is thought to be one of the mechanisms involved in attenuated salt-induced sympathoexcitation caused by miso intake.

Reduction of oxidative stress may be another key player in the attenuated salt-induced sympathoexcitation caused by miso intake. The decrease in brain oxidative stress due to miso intake was reported in a study using Dahl-S rats [56]. Brain oxidative stress is well known to be associated with blood pressure elevation via sympathoexcitation [57–59]. Furthermore, there is the complex interaction between RAS, oxidative stress, and SNA. For example, oxidative stress generated by RAS activation in the brain is reported to contribute to sympathoexcitation and the pathogenesis of cardiovascular disease [60]. Therefore, reduction of both RAS activity and oxidative stress in the brain may be involved in attenuated salt-induced sympathoexcitation in response to habitual dietary intake of miso soup.

## Effects of miso on other molecular mechanisms which regulate blood pressure

The attenuation of the salt-induced blood pressure elevation by miso intake may be caused by vasodilation and natriuresis independent of SNA. In fact, it was reported that RAS activation causes vasoconstriction through enhanced myosin light chain kinase expression [61]. In addition, previous studies revealed the relationship between RAS and vascular injury characterized by endothelial dysfunction, structural remodeling, and inflammation [62]. Oxidative stress is also associated with the pathogenesis of hypertension via the deterioration of vascular function [63, 64]. Furthermore, the RAS in the kidney is also known to contribute to blood pressure regulation via modulation of sodium homeostasis. Specifically, AT1R expression in the renal proximal tubule is reported to regulate sodium homeostasis. Increases in AT1R expression in the proximal tubule leads to blood pressure elevation [65]. Oxidative stress also contributes to renal tubular transport including sodium transport [66]. There are complex relationships between RAS and oxidative stress in various organs that are involved in blood pressure regulation. Therefore, the reduction of both RAS activity and oxidative stress in several organs, such as the brain, blood vessels, or kidney, may be involved in the attenuated salt-induced hypertension following the habitual dietary intake of miso soup.

## Perspectives

This review article summarizes the effects of miso intake on SNA, blood pressure, and heart rate. Because miso contains some amount of salt, habitual miso intake is expected to increase blood pressure. However, previous basic studies clearly demonstrated that miso intake attenuates salt-induced sympathoexcitation and blood pressure elevation in the salt-sensitive hypertensive models. In addition, it was also demonstrated that miso intake lowers SNA in normal models. Basic animal experiments have shown the direct effects of miso intake on SNA, blood pressure, and heart rate. Collectively, these findings support that miso has a potential to lower SNA, blood pressure, and heart rate.

The results obtained from animal experiments cannot be directly applied to clinical settings. Previous clinical studies failed to demonstrate the relationship between the frequency of miso intake and blood pressure levels. However, the heart rate, which is a useful marker of SNA, was lower in subjects with frequent miso soup consumption than in those with infrequent miso soup consumption, particularly in the winter. This may mean that the results of clinical studies and basic studies do not contradict each other. However, data are inadequate to conclude the effects of miso on blood pressure and heart rate in humans. The subjects with high miso soup consumption are thought to have the traditional Japanese dietary pattern. If so, they may consume more vegetables, seaweeds, and fish as well as miso. Therefore, the beneficial effects observed in the subjects with high miso soup consumption may be a result of not only miso, but also other foods. As such, interventional studies are necessary to establish the effects of miso on blood pressure and heart rate in human. In addition, there are other concerns with the interpretation of the findings of the previous studies. First, the precise mechanisms involved in the lowering of SNA, blood pressure, and heart rate following miso intake have not been fully understood. The miso ingredients that have inhibitory effects on SNA, blood pressure, and heart rate remain unclear. Second, the appropriate dietary intake of miso remains unclear. Third, previous studies demonstrated the safety of habitual miso soup intake in subjects with normotension or stage I hypertension, but not in those with stage II or III hypertension. Also, the safety of habitual miso intake in subjects with significant salt sensitivity remains unclear. Further studies will be needed to clarify these points.

## Conclusion

The intake of miso soup does not increase blood pressure and heart rate compared with the equivalent intake of salt, probably in part due to the lowering of SNA.

## Data Availability

Not applicable

## Abbreviations

ACE: Angiotensin-converting enzyme
aCSF: Artificial CSF
AT1R: Angiotensin type 1 receptor
CPO: Chronic pressure overload
CSF[Na]: Sodium concentration in the cerebrospinal fluid
Dahl-S rats: Dahl salt-sensitive rats
ECG: Electrocardiogram
HF: High frequency
ICV: Intra-cerebroventricular
IP: Intraperitoneal
LF: Low frequency
MR: Mineralocorticoid receptor
RAS: Renin-angiotensin system
Sham-mice: Sham-operated control mice
SHR: Spontaneously hypertensive rats
SNA: Sympathetic nerve activity
uNa: Urinary sodium
uNE: Urinary norepinephrine
